# Comparison of actigraphy with a sleep protocol maintained by professional caregivers and questionnaire-based parental judgment in children and adolescents with life-limiting conditions

**DOI:** 10.1186/s12904-024-01394-7

**Published:** 2024-02-23

**Authors:** Larissa Alice Kubek, Benedikt Claus, Boris Zernikow, Julia Wager

**Affiliations:** 1 PedScience Research Institute, Datteln, Germany; 2 Department of Children’s Pain Therapy and Paediatric Palliative Care, Witten/Herdecke University,Faculty of Health, School of Medicine, Witten, Germany; 3 Paediatric Palliative Care Centre, Children’s and Adolescents’ Hospital, Datteln, Germany

**Keywords:** Chronic disease, Pediatrics, Palliative care, Actigraphy, Diagnostic techniques and procedures

## Abstract

**Background:**

Actigraphy offers a promising way to objectively assess pediatric sleep. Aim of the study was investigating the extent to which actigraphy used in children and adolescents with life-limiting conditions is consistent with two other measures of sleep diagnostics.

**Methods:**

In this monocentric prospective study *N* = 26 children and adolescents with life-limiting conditions treated on a pediatric palliative care unit were assessed. For three consecutive nights they wore an actigraph; the 24-hours sleep protocol documented by nurses and the Sleep Screening for Children and Adolescents with Complex Chronic Conditions (SCAC) answered by parents were analyzed. Patient characteristics and the parameters sleep onset, sleep offset, wake after sleep onset (WASO), number of wake phases, total sleep time (TST) and sleep efficiency (SE) were descriptively examined. Percentage bend correlations evaluated the three measures’ concordance.

**Results:**

Descriptively, and except for the number of waking episodes, the different measures’ estimations were comparable. Significant correlations existed between actigraphy and the sleep protocol for sleep onset (r = 0.83, p = < 0.001) and sleep offset (r = 0.89, p = < 0.001), between actigraphy and SCAC for SE (r = 0.59, *p* = 0.02).

**Conclusion:**

Agreement of actigraphy with the focused sleep measures seems to be basically given but to varying degrees depending on the considered parameters.

**Supplementary Information:**

The online version contains supplementary material available at 10.1186/s12904-024-01394-7.

## Introduction

Of the approximately 21 million children worldwide deemed in need of a pediatric palliative care approach, many present with a variety of heterogeneous and interrelated symptoms [1, 2]. One of these symptoms, with a prevalence rate of around 50–70%, is sleep problems [3–6]. Sleep problems can be highly burdensome both physically and psychologically for the child and the family affected due to their often refractory nature and their accumulated occurrence [7–9]. For instance, the sleep problems experienced by affected children and adolescents can adversely affect their everyday life and daytime functioning, potentially leading to reduced quality of life [8, 10]). Conversely, many parents report experiencing sleep problems themselves, along with irritability, high stress levels, exhaustion, or head pain [11, 12]. Furthermore, as sleep problems can arise from a wide variety of causes and may, for example, be an expression of other symptoms such as pain, monitoring sleep patterns is crucial for understanding a child’s overall situation and current challenges [2, 13, 14]. Optimal treatment indications and adequate therapy monitoring can thus be supported. Therefore, adequate diagnosis poses the important basis for the initiation of potential therapeutic options [7–9]. As parents become experts on their child over the years, their assessments that can be easily and cost-effectively obtained using questionnaires such as the recently developed Sleep Screening for Children and Adolescents with Complex Chronic Conditions (SCAC) represent an important pillar of diagnostics [3, 7, 15].

A promising, more objective and relatively low cost diagnostic measure is provided by so-called actigraphs [16]. These resemble a modern sports watch and are usually worn on the wrist or ankle and can thus theoretically be used in any environment. The motion sensor in the actigraph allows differentiation between movement and lack of movement and, on this basis, enables statements to be made about the wearer’s sleep and wakefulness [7]. Compared to the gold standard polysomnography (PSG), actigraphy has the considerable advantage that it can be utilized in almost any setting and is less burdensome for the child and their family. For example, there is no need for the child to spend a night in the unfamiliar surroundings of a sleep laboratory [7, 17]. Actigraphy has been recognized by the American Academy of Sleep Medicine, among others, as a helpful clinical tool that can non-invasively generate a variety of relevant parameters across a wide range of disorders. Clinical decisions can be supported, and therapy trials monitored using the device [18]. Actigraphy is unobtrusive and practical, as it can be used over any period of the day or night [19]. In addition, there is evidence that actigraphy can generate reliable sleep data in children with life-limiting conditions [20].

Through research with healthy children and children with complex chronic conditions such as epilepsy [21], it appears that there is uncertainty about the extent to which actigraphy data are consistent or inconsistent with data from other (subjective) diagnostic measures [22, 23]. Appropriate studies with a heterogeneous group of children with life-limiting conditions are to our best knowledge non-existent. The aim of this study therefore was examining the extent to which actigraphy data from a sample of children and adolescents with pediatric palliative care needs and life-limiting conditions are consistent with a third-party sleep screening tool (SCAC) and the 24-hours sleep protocol developed at the Pediatric Palliative Care Center of the Children’s and Adolescents’ Hospital Datteln – Witten/Herdecke University. Such an investigation can yield important insights into the correspondence of sleep parameters across different measurement instruments and identify which parameters are best assessed by each instrument.

## Methods

For reporting the research methods and findings of this study, the STROBE checklist of the Equator network (http://www.equator-network.org/library/) was used.

### Participants

For the investigation of the study question, eligible participants were children and adolescents with life-limiting conditions and an age between 1 and 25 years, who were inpatients on a pediatric palliative care unit of a pediatric tertiary hospital in Datteln, Germany [24]. This age range was chosen considering that many pediatric palliative care patients experience developmental delays. Consequently, they are still considered “pediatric” and receive treatment on pediatric units, even if their biological age exceeds 18 years [25]. Exclusion criteria were defined as insufficient local language skills of the parents and an acute medical or psychological crisis of the child or the family. The study was approved by the Ethics Committee of Witten/Herdecke University (approval code: 128/2020, approval date: August 15, 2020). Because all of the participating children and adolescents were incapable of giving informed consent themselves due to their conditions and associated cognitive impairments, this was given vicariously by the parent who accompanied the child during their inpatient stay (96.2% mother, 3.3% father).

### Measurements

Different objective and subjective measures were used to record children’s and adolescents’ sleep behavior. All instruments focused on a reference period of three consecutive nights. This duration was determined as the optimal compromise between having a sufficiently long recording time to generate meaningful sleep data and ensuring practicability for the clinical context (e.g. considering average inpatient lengths of stay). In addition, this reference period aligns with official recommendations for the (minimum) duration of (actigraphy) studies [26]:

- Actigraphy by means of the Actiwatch 2 (Philips Respironics, Murrysville, PA) that was put around the children’s/adolescent’s wrist (right or left side by default, as a dominant hand could not be reliably determined in patients due to their underlying conditions). For the devices’ configuration and data analysis, the manufacturer’s accompanying Actiware Software package (Philips Respironics, Murrysville, PA; version 6.1.2) was used. Activity data were recorded in 15-seconds epochs. For establishing the wake phases, the medium sensitivity level offered by the manufacturer was chosen (requirement of 40 activity counts before an epoch is considered awake [27]; already shown that this level is adequate for most sleep parameters [20]).
- An adapted version of the recently developed Sleep Screening for Children and Adolescents with Complex Chronic Conditions (SCAC [15];). Other than in the original version, a reference period of 3 days (in the sense of “How often did your child during the last 3 days...?) instead of 7 days was used (see 1.3 Data collection). In addition, items were added to record basic sleep information (e.g., time of falling asleep, time of waking up; Supplemental Material 1). These adaptations enabled the later comparability of the focused sleep measures respectively its generated sleep parameters. To ensure that this adapted version was comparable to the original version of the SCAC, a pretest was conducted (see 2.3 Data collection and following sections).
- The 24-hours sleep protocol. This was developed at the Pediatric Palliative Care Center Datteln, Germany, to systematically record inpatient children’s or adolescents sleep and wake times throughout the 24-hour day in a 15-minute grid (Additional file 1 [28];). An important advantage of the 24-hours sleep protocol compared to sleep logs commonly used in sleep medicine is that the former is not kept retrospectively after the night experienced, but practically in real time and without omissions. Any missing entries are promptly detected and completed during the respective care handovers [29]. In addition, events such as irritability, pain, or medication/ food intake can be documented. The protocol is routinely completed by nurses on duty during their shifts and therefore did not involve any additional work for the practitioners in the context of the study Since the 24-hours sleep protocol generates factual/objective data and can therefore be legitimately kept and analyzed over any desired period of time, a pretest for the instrument’s 3-day assessment was not necessary.
- The children’s and adolescents’ care reports, which are written by the unit’s nurses. From this, especially the children’s and adolescents time of falling asleep was used as a reference for the evaluation of the actigraphy data (see 2.4 Data analysis).

### Data collection

For the pretest (validation of the adapted SCAC), parents of children and adolescents with life-limiting conditions treated at the Children’s and Adolescents’ Hospital Datteln were recruited (March–April 2021). If parents consented to participate in the study, they completed the SCAC original version (reference period 7 days) and the adapted version (reference period 3 days) at admission. Across all participants, the order of the two forms was randomized (original first, then adaptation vs. adaptation first, then original).

For the main study, parents of children and adolescents admitted to the pediatric palliative care unit who met the inclusion criteria were provided with comprehensive verbal and written information about the study. Upon request for participation, the actigraph was configured by the study coordinator (L.A.K.), taken back to the unit and given to the nurses. On each of the three consecutive survey evenings, they applied the actigraph to the child. An explicit time for this was not specified but oriented to the individual child’s rhythm (see 2.4 Data analysis). The SCAC was filled out by parents the morning after the third assessment night, so that the questionnaire’s reference period coincided with the actigraphy period. The actigraphy data were retrieved and analyzed. The children’s/adolescents’ 24-hours sleep protocol and the children’s/adolescents’ care reports for the three assessment nights were also reviewed and analyzed.

### Statistical analysis

In the pretest, the items of both questionnaire versions were compared using Spearman-Rho correlation. Randomization effects were examined using a Mann-Whitney-U test.

In the main study, actigraphy data were read out by the study coordinator and analyzed using Actiware®. This software package is tailored specifically to the Actiwatch 2, enabling us to configure and access the device, as well as manage, analyze, and export the recorded activity data. Actiware and Actiwatch 2 are designed for use with both pediatric and adult patients, and have been employed in both populations [22, 30–33]. To find a child’s rest interval (period during which the child rests and is less active), the care reports were considered. From the information given there about a child’s sleep onset time, 30 minutes were calculated back to define the start of the rest interval. To the indication of when a child woke up, 30 minutes were added to care report information define the end of the rest interval. This procedure has already been presented in the literature when clear times for the start and end of an actigraphy assessment cannot be clearly delineated without a reference document, as in our case [34]. By defining a rest interval, the child’s sleep and wake times were detected by the automatic logarithm of the Actiware®. The following sleep parameters were extracted: sleep onset time (the first 10 immobile minutes within the rest interval), sleep offset time (the last 10 immobile minutes within the rest interval), wake after sleep onset (WASO), total number of wake phases, total sleep time (TST), and sleep efficiency (SE; defined as the actual time spent sleeping during the night; specified in percent). These sleep parameters are standard parameters in sleep medicine practice and research [35]. In comparing sleep measures, we extracted all relevant parameters from each instrument that could be reliably generated.

For the 24-hours sleep protocol, the same parameters as for actigraphy were evaluated except for SE. This was because bedtime in particular, which is crucial for determining sleep efficiency, is not routinely recorded by clinicians in the protocol. If this information is noted, it is usually included in the care report to avoid duplicate documentation in both documents. Therefore, since it was intended to evaluate the 24-hours sleep protocol unaltered in its usual form, SE was not generated accordingly. (). In the SCAC, the parameters sleep onset and SE were considered. The other parameters were neglected in view of the consideration that parents are not at their child’s side during the nights on the pediatric palliative care unit and thus per se cannot generate reliable statements on these aspects. For the 24-hours sleep protocol, average values were first calculated from the three nights under consideration. Patient characteristics and the three diagnostic measures’ sleep parameters considered are presented by means of descriptive statistics. The three measures’ relationship was determined using percentage bend correlation. In this non-parametric procedure, ß = 10% of the largest and smallest values are not taken into account, allowing better handling of outliers [36]. Multiple comparisons were adjusted via false discovery rate correction (FDR, [37]). All analyses were performed using SPSS (IBM, version 28) and R [38] with RStudio [39].

## Results

### Pretest

*N* = 30 parents participated in the pretest (*n* = 16 in the sequence: adaptation (3-day reference time frame) vs. original condition (7-day reference time frame); *n* = 14 in the sequence: original vs. adaptation condition). In *n* = 27 (90%) of the cases, the child’s mother completed the questionnaire. 92.2% (*n* = 26) stated that they were the child’s primary caregiver. Participants (mean age *M* = 8.61 years, range: 1–18 years) were 63.3% (*n* = 19) male and 36.7% (*n* = 11) female. 96% of the examined items (range: *r* = 0.46–1.00) showed a strong correlation relationship between the 3- and 7-day reference time frame and 4% a moderate relationship (r = 0.46, r = 0.49) with the respective items of the other version [40]. There was no significant difference between the two questionnaire versions (Mann-Whitney U test, *p* > 0.05; range: *p* = 0.07–1.00). From the results it was concluded that the adapted version of the SCAC was plausibly applicable for the main test.

### Main study

*N* = 29 children and adolescents were included in the main study. The data sets of *n* = 3 children had to be excluded due to technical errors in the actigraphy assessment, so that the final sample consisted of *N* = 26 children and adolescents. The participants’ characteristics are shown in Table 1.

**Table 1:** Characteristics of children and adolescents participating in the main test (*N* = 26)

Age in years; *M* (*SD*)		9.92 (6.3)
Age groups; *n* (%)		
1–5		7 (26.9)
6–10		7 (26.9)
11–15		6 (23.0)
16–20		4 (15.3)
21–25		2 (7.6)
Sex; *n* (%)		
Female		11 (42.3)
Male		15 (57.7)
Born premature; *n* (%)		
Yes		4 (18.2)
Care level; *n* (%)^1^		
None		1 (3.8)
1		0 (0)
2		0 (0)
3		1 (3.8)
4		5 (19.2)
5		19 (73.1)
Can the child communicate verbally?; *n* (%)		
Yes, without restrictions		2 (9.1)
Yes, with restrictions		3 (13.6)
No		17 (77.3)
Diagnoses^2^		
ICD-10 code	Definition of ICD 10 code and examples of underlying disease(s)	number (%)
P91	Other disturbances of cerebral status of newborn	9 (34.6)
	*Hypoxic ischemic encephalopathy, Periventricular leukomalacia*	
Q87	Other specified congenital malformation syndromes affecting multiple systems	5 (19.2)
	*Microdeletion syndrome 4q13.3–21.3, mosaic trisomy 9*	
Q04.9	Congenital malformation of brain, unspecified	4 (15.4)
	Severe psychomotor developmental disorder with bifrontal polymicrogyria and hypomyelination.	
E70-E70	Metabolic disorders	3 (11.5)
	*Metachromatic leukodystrophy*	
Q93.9	Disorder of brain, unspecified	2 (7.7)
	*Epileptic encephalopathy*	
G71	Primary disorders of muscles	1 (3.8)
	*X-linked recessive muscular dystrophy, Duchenne type*	
Q89	Other congenital malformations, not elsewhere classified	1 (3.8)
	*Severe global developmental disorder caused by mutation in the ACY1 gene.*	
G93.1	Anoxic brain damage, not elsewhere classified	1 (3.8)
	*Severe multi-cystic encephalomalacia*	

^1^In Germany, the care level indicates the degree to which a person requires care ranging from 1 (slight impairment of independence) to 5 (most severe impairment of independence with special requirements for nursing care)

^2^Examples of the represented underlying diseases are indicated in italic font; multiple diagnoses/entries possible

#### Interrelationships of the three sleep measures

**Fig. 1:**
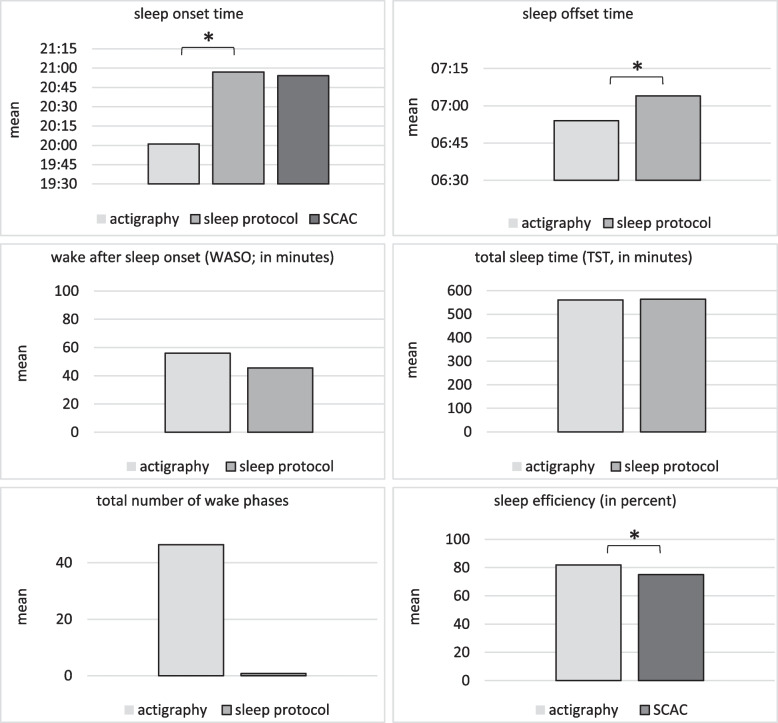
Descriptive presentation of the sleep parameters assessed in the diagnostic measures actigraphy, 24-hours sleep protocol, and Sleep Screening for Children and Adolescents with Complex Chronic Conditions (SCAC)

Descriptively, according to the 24-hours sleep protocol, the children and adolescents fell asleep on average about 1 hour and according to the parents about 45 minutes later than according to the actigraphy. Also, the patients studied woke up on average about a quarter of an hour later when measured by the 24-hour sleep protocol than when measured by the actigraph. The actigraph descriptively showed a longer average WASO although the descriptive difference from the sleep protocol in this case was only about 10 minutes. For the TST, both measures considered showed a roughly similar descriptive picture The clearest descriptive difference between the 24-hours sleep protocol and actigraphy was toward the parameter of total number of wake phases. Children’s/adolescents’ SE was descriptively rated slightly higher by actigraphy than by parents in the SCAC. The mean values of the three sleep measures actigraphy, 24-hours sleep protocol and SCAC for the investigated sleep parameters are shown in Fig. 1 and Table 2.

**Table 2:** Mean values of the three sleep measures actigraphy, 24-hours sleep protocol and the SCAC

	Actigraphy, *M* (*SD*)	24-hours sleep protocol, *M* (*SD*)	SCAC, *M* (*SD*)
Sleep onset	20:01 (4:05)	20:57 (1:06)	20:54 (1:32)
Sleep offset	6:54 (1:35)	7:04 (1:16)	ND
Total sleep time (TST)	560.56 (120.95)	563.57 (92.58)	ND
Wake after sleep onset (WASO)	55.92 (52.69)	45.6 (52.77)	ND
Number of wake phases	46.39 (29.33)	0.79 (0.75)	ND
Sleep efficiency (SE)	81.93 (15.04)	ND	75.04 (17.56)

*p < 0.05. **p < 0.01

*ND* parameter not determined

**Fig. 2:**
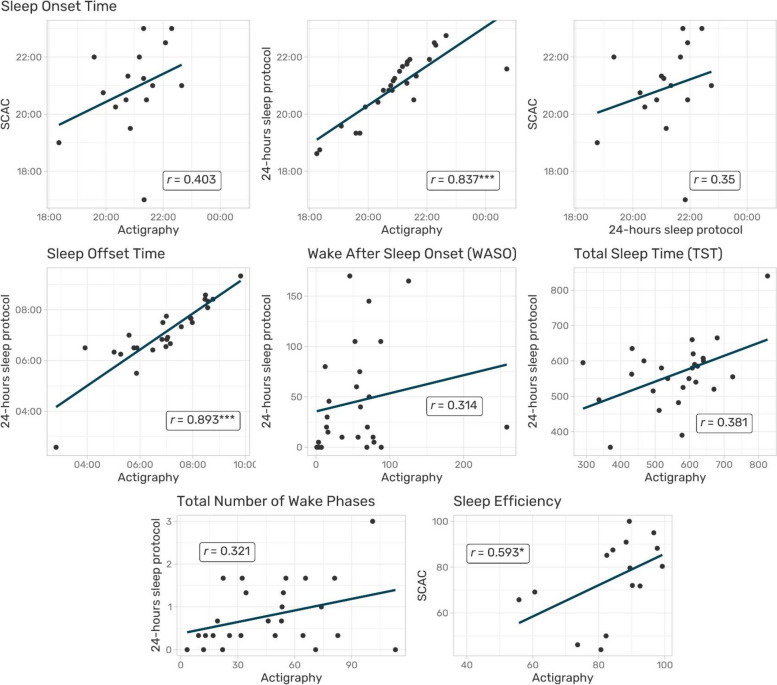
Scatter plots and statistics of the correlations between actigraphy, 24-hour sleep protocol and SCAC with regard to the examined sleep parameters

Strong positive correlations were found for the parameters sleep onset (*r* = 0.83, p = < 0.001) and sleep offset (*r* = 0.89, *p* = < 0.001) between the 24-hours sleep protocol and actigraphy. Another positive correlation existed between the SCAC and actigraphy in terms of children’s/adolescents’ SE *(r =* 0.59, *p* = 0.02; Fig. 2).

## Discussion

### Results of the study

The present study aimed to demonstrate the extent to which actigraphy is consistent with other diagnostic measures, particularly the objective 24-hours sleep protocol, in children and adolescents with life-limiting conditions.

With regard to sleep onset time and sleep offset time, it was shown that the two measures yielded significantly similar results. Since delayed sleep onset times resulting from problems with sleep initiation are common in this population, it is important that this information can be reliably recorded during diagnostics in the clinical setting [32, 41–43]. This, in turn, appears to be the case for both instruments. It is worth mentioning here that established diagnostic systems such as the International Classification of Sleep Disorders speak of a delay in falling asleep from > 20 minutes after bedtime; instruments such as actigraphy or the sleep protocol, therefore, do not have to be able to depict “standard values” as to when a child of the corresponding (biological) age ought to fall asleep [44]. Although no significant correlations between the measurements of the actigraphy and the sleep protocol could be found for the two further parameters “wake after sleep onset” (WASO) and “total sleep time” (TST), the descriptive comparisons nevertheless revealed comparable scores for both measures.

Basically, these findings indicate that both actigraphy and the 24-hours sleep protocol are suitable to objectively record specific sleep parameters in critically ill children and adolescents. Observation protocols such as the sleep protocol require active processing by a facility’s staff (usually nurses). At the same time, such protocols in the form of sleep diaries are also often filled out by parents in order to provide diagnosticians with a child’s relevant sleep information [3, 42, 45]. However, a significant disadvantage of sleep protocols involves the fact that they represent a not inconsiderable additional effort for the person completing them. For example, nurses are required to complete them during their shift, leaving them less time for the patient and their other tasks. For parents, such a duty can be stressful, since caring for their child is already time-consuming per se, and the child’s sleep problems often lead to health problems for the parents as well [46, 47]. At this point, actigraphy may serve as a tool with great potential: once configured, sleep information is collected without a person’s further intervention. Professionals and parents could be relieved by this and devote themselves to other important activities. Biases, for example due to misremembered facts, or gaps due to non-documentation can be prevented [48]. The relevance of the first aspect is also illustrated by the parameter of the number of nocturnal wake phases considered in our study.

Descriptively, the largest difference between the actigraphy and the sleep protocol was found for this parameter. A first possible explanation could be that the nursing staff completing the 24-hours sleep protocol are not continuously at the patient’s bedside during the night and therefore do not detect every wake phase. Another, perhaps even more likely approach is the very different approaches of the instruments for detecting waking phases: The complex technical logarithm of actigraphy allows for the identification of each individual phase of a child’s wakefulness. Moreover, these devices have the capability to reliably detect waking responses that might go unnoticed by an external observer or even by the individual themselves [49]. Observation instruments such as the 24-hours sleep protocol, on the other hand, presume that the focused behavior is actually noticed. Even if the nursing staff in our study had been present in the patient’s room around the clock, they would probably not necessarily have recognized the nocturnal waking phases as registered by actigraphy [50].

The objective and reliable recording of nocturnal awakenings is important for pediatric palliative patients as well as for healthy individuals, given its prevalence as a common concern [51–53]. Interestingly, our observations on the frequency of night-time awakenings also align with findings in healthy children and those with less serious conditions such as autism [50, 53, 54]. A trend emerges, indicating that parents or subjective assessors are worse at estimating nocturnal awakenings than objective measures, relatively independent of their child’s specific (medical) situation. Disrupted sleep poses enormous stress for both the children and their parents and may contribute to diminished daytime performance and negative mood, among other effects [55]. On one hand, actigraphy, when used independently, exhibits considerable potential for objectively recording sleep interruptions, a facet that subjective assessments may not reliably capture [45]. In addition to waking up at night “by themselves”, many children and adolescents with life-limiting conditions also experience nocturnal sleep interruptions due to caregiving activities such as administering medication, feeding, or repositioning [47]. Distinguishing spontaneous awakenings from those induced by caregiving is essential for effective sleep diagnostics and subsequent therapeutic interventions. In this context, the combination of actigraphy with other observational instruments can be beneficial. These instruments offer detailed insight into the nature and frequency of (nursing) interventions conducted during the night, with actigraphy providing objectivity and facilitating the nuanced assessment and differentiation of the reasons behind nocturnal sleep awakenings [56].

The question of a child’s sleep efficiency can apparently be equally reliably answered by actigraphy as by the parents. Basically, having several reliable ways to determine this parameter is very welcomed, as many of the young patients show reduced sleep efficiency, especially with increasing disease severity [31, 57, 58]. In addition, depending on the specific setting (home, clinical), the instrument that is best suited to the specific situation (of the family) and question can be selected. At first glance, however, our findings contradict the idea that parents often misjudge their child’s sleep, especially if they do not sleep in the child’s room [45]. It is possible, however, that parents are able to provide reliable information on more global parameters such as sleep efficiency, whereas they struggle with the assessment of very specific parameters such as the number of wake phases. Since we did not consider corresponding specific parameters from the parents’ point of view in this study, this should be the content of further studies. Also, the parents’ statements regarding these parameters should be compared with data from polysomnography in order to be able to make statements about its reliability.

Overall, the findings on the SCAC variables primarily reinforce that this questionnaire is an internally consistent and versatile instrument. This is evident by a good fit with the data acquired by actigraphy. This is encouraging, as there are only a few instruments that have been developed specifically for these highly complex patients [15, 59]. Sleep questionnaires frequently used in research and literature have typically been developed for healthy or less impaired children and adolescents, overlooking the particularities and special needs of young patients with life-limiting conditions [15, 59]. Actigraphy was recently shown to be an apparently reliable measure in children and adolescents with life-limiting conditions and severe neurological impairment when compared with the gold standard PSG, so it may be considered the gold standard in this study ( [20, 42]). What might account for the lack of agreement between the information obtained by actigraphy and the 24-hours sleep protocol and the SCAC scales should be evaluated in follow-up studies. Perhaps differing results will emerge with larger sample sizes. Another explanation could be that the scales of a subjective instrument such as the SCAC account for fundamentally different aspects than the other two diagnostic measures considered, so that agreement cannot be assumed for these specific instruments [18]. Clearer findings in this regard could help to decide when and which subjective or objective instrument should be usefully employed during the diagnostic process in order to be able to gather the most reliable information possible about a child’s sleep.

### Limitations of the study

The study’s major limitation is the comparatively small sample size. This prevented more complex analyses that could have supported a clearer interpretation of the results. In addition, for various comparisons of the parameters and instruments, we could only report descriptive similarities. Nevertheless, to the best of our knowledge, this is the first study in the highly vulnerable setting of pediatric palliative care that focused in parallel on different diagnostic measures including actigraphy and children and adolescents with heterogeneous clinical pictures. In this sense, this study is to be considered as a prelude for further follow-up studies which should further clarify which role actigraphy can play in the future for the diagnosis of sleep problems in children and adolescents with life-limiting conditions, respectively which diagnostic measures can be meaningfully combined in the future to obtain the most comprehensive picture of a child’s sleep.

Another limitation of the study is that we did not test the data of the SCAC and 24-hours sleep protocol against the gold standard of sleep diagnostics polysomnography and thus cannot say which measure provided the most correct results. Validations such as this have become increasingly common for various instruments in recent years, but a corresponding comprehensive comparison in the population of children and adolescents with life-limiting conditions is lacking [23, 60, 61]. Future studies should build on our kick-off project to provide the highest quality sleep diagnostics in the long term. To achieve this, collaborations between different clinics and sleep laboratories could be sought.

Because parents of children and adolescents receiving care in the pediatric palliative care unit typically do not sleep in their children’ room at night, not all sleep parameters could be determined from the parents’ perspective. It has already been pointed out that correct conclusions about children’s/ adolescents’ nighttime sleep from subjective measures such as parental testimony can be fundamentally difficult to obtain and prone to error [17, 45]. Nevertheless, parents are the experts for their child and important informants about the sleep behavior [3]. Our results also indicate that certain parameters may well be reliably reflected by parents. This consideration should definitely be pursued in the future and in larger studies. For this purpose, data collection could be shifted to the children’s or adolescents home setting, where parents more often spend the night with their child.

A weakness of the 24-hours sleep protocol in our study is the inability to determine SE using the core information in the document. However, this limitation may not necessarily apply to all settings. In institutions where a child’s bedtime routine is primarily managed by clinicians and administrative decisions mandate the inclusion of this time in the 24-hours sleep protocol, the SE parameter could be generated. This circumstance shows quite impressively that, unlike validated questionnaires, such observation instruments are flexible in their specific application. This flexibility can be both an advantage and a disadvantage. In our study, we aimed to evaluate the instrument in its natural usage within the pediatric palliative care unit. In future research, especially multicenter projects, establishing uniform rules for completing the instrument, at least for the study period, is essential to ensure optimal comparability of results. In this study, actigraphy was compared for the first time in a heterogeneous sample of pediatric palliative care with two other measures commonly used in sleep diagnostics. Although the study should be seen as a prelude to further larger projects, the results indicate that a) while actigraphy is an apparently reliable tool in this sample, sleep information can also be effectively obtained by other measures, which may be faster and technically easier to use, and b) the generation of reliable data depends on the specific parameter and instrument considered.

### Supplementary Information

**Supplementary Material 1.**

**Supplementary Material 2.**

## Data Availability

Anonymized data are available from the corresponding author upon reasonable request.

## References

[1] Connor SR, Downing J, Marston J (2017). Estimating the global need for palliative Care for Children: a cross-sectional analysis. J Pain Symptom Manag..

[2] Hoell JI, Weber H, Warfsmann J, Trocan L, Gagnon G, Danneberg M (2019). Facing the large variety of life-limiting conditions in children. Eur J Pediatr..

[3] Schwantes S, O'Brien HW (2014). Pediatric palliative care for children with complex chronic medical conditions. Pediatr Clin N Am..

[4] Malcolm C, Forbat L, Anderson G, Gibson F, Hain R (2011). Challenging symptom profiles of life-limiting conditions in children: a survey of care professionals and families. Palliat Med..

[5] Hauer JM, Wolfe J (2014). Supportive and palliative care of children with metabolic and neurological diseases. Curr opinion support palliative care..

[6] Stangenes KM, Fevang SK, Grundt J, Donkor HM, Markestad T, Hysing M, et al. Children born extremely preterm had different sleeping habits at 11 years of age and more childhood sleep problems than term-born children. Acta paediatrica Oslo, Norway 2017;106(12):1966–1972. 10.1111/apa.13991 28714101

[7] Simard-Tremblay E, Constantin E, Gruber R, Brouillette RT, Shevell M (2011). Sleep in children with cerebral palsy: a review. J Child Neurol..

[8] Tietze AL, Zernikow B, Michel E, Blankenburg M (2014). Sleep disturbances in children, adolescents, and young adults with severe psychomotor impairment: impact on parental quality of life and sleep. Dev Med Child Neurol..

[9] Blackmer AB, Feinstein JA (2016). Management of Sleep Disorders in Children With Neurodevelopmental Disorders: A Review. Pharmacotherapy..

[10] Ghorbanpour Z, Hosseini SA, Akbarfahimi N, Rahgozar M (2019). Correlation between sleep disorders and function in children with spastic cerebral palsy. Iranian j child neurol..

[11] Morelius E, Hemmingsson H (2014). Parents of children with physical disabilities - perceived health in parents related to the child's sleep problems and need for attention at night. Child Care Health Dev..

[12] Didden R, Korzilius H, van Aperlo B, van Overloop C, de Vries M (2002). Sleep problems and daytime problem behaviours in children with intellectual disability. J intellect disab res: JIDR..

[13] Dreier LA, Wager J, Blankenburg M, Zernikow B. The unfavorable Alliance of pain and poor sleep in children with life-limiting conditions and severe psychomotor impairment. Children (Basel, Switzerland). 2018;5(7). 10.3390/children5070082 PMC6068563 29933542

[14] Marshansky S, Mayer P, Rizzo D, Baltzan M, Denis R, Lavigne GJ (2017). Sleep, chronic pain, and opioid risk for apnea. Prog Neuro-Psychopharmacol Biol Psychiatry.

[15] Kubek LA, Claus B, Rostasy K, Bertolini A, Schimmel M, Frühwald MC, et al. Development and preliminary validation of the sleep screening for children and adolescents with complex chronic conditions (SCAC). J Sleep Res. 2023:e13881. 10.1111/jsr.13881. 10.1111/jsr.13881 36929532

[16] Smith MT, McCrae CS, Cheung J, Martin JL, Harrod CG, Heald JL (2018). Use of Actigraphy for the evaluation of sleep disorders and circadian rhythm sleep-wake disorders: an American Academy of sleep medicine systematic review, Meta-analysis, and GRADE assessment. J clin sleep med: JCSM : official public American Academ Sleep Med..

[17] Combs D, Goodwin JL, Quan SF, Morgan WJ, Hsu C-H, Edgin JO (2019). Mother knows best? Comparing child report and parent report of sleep parameters with polysomnography. J clin sleep med: JCSM : official public American Academ Sleep Med..

[18] Smith MT, McCrae CS, Cheung J, Martin JL, Harrod CG, Heald JL (2018). Use of Actigraphy for the evaluation of sleep disorders and circadian rhythm sleep-wake disorders: an American Academy of sleep medicine clinical practice guideline. J clin sleep med: JCSM : official public Am Academy Sleep Med..

[19] Dayyat EA, Spruyt K, Molfese DL, Gozal D (2011). Sleep estimates in children: parental versus actigraphic assessments. Nat sci sleep..

[20] Kubek LA, Kutz P, Roll C, Zernikow B, Wager J. Applicability of Actigraphy for assessing sleep behaviour in children with palliative care needs benchmarked against the gold standard polysomnography. J Clin Med. 2022;11(23) 10.3390/jcm11237107 PMC9739292 36498681

[21] Feudtner C, Feinstein JA, Zhong W, Hall M, Dai D (2014). Pediatric complex chronic conditions classification system version 2: updated for ICD-10 and complex medical technology dependence and transplantation. BMC Pediatr..

[22] Siegel BI, Cakmak A, Reinertsen E, Benoit M, Figueroa J, Clifford GD (2019). Use of a wearable device to assess sleep and motor function in Duchenne muscular dystrophy. Muscle Nerve.

[23] Niel K, LaRosa KN, Klages KL, Merchant TE, Wise MS, Witcraft SM, et al. Actigraphy versus polysomnography to measure sleep in youth treated for Craniopharyngioma. Behav Sleep Med. 2019:1–9. 10.1080/15402002.2019.1635133 31303059

[24] Jaworska N, MacQueen G (2015). Adolescence as a unique developmental period. J psychiat neurosci: JPN..

[25] Benini F, Papadatou D, Bernadá M, Craig F, De Zen L, Downing J (2022). International standards for pediatric palliative care: from IMPaCCT to GO-PPaCS. J Pain Symptom Manag..

[26] Littner M, Kushida CA, Anderson WM, Bailey D, Berry RB, Davila DG (2003). Practice parameters for the role of actigraphy in the study of sleep and circadian rhythms: an update for 2002. Sleep..

[27] Toon E, Davey MJ, Hollis SL, Nixon GM, Horne RS, Biggs SN (2016). Comparison of commercial wrist-based and smartphone accelerometers, Actigraphy, and PSG in a clinical cohort of children and adolescents. J clin sleep medicine : JCSM : official public Am Academy Sleep Med..

[28] Pediatric Palliative Care Center. 24-hours sleep protocol, Datteln, Germany (2024). Available from: https://usercontent.one/wp/kinderpalliativzentrum.de/wp-content/uploads/2018/02/24-stunden-protkoll.pdf?media=1686745038.

[29] Meltzer LJ, McLaughlin CV (2015). Pediatric sleep problems: a clinician's guide to behavioral interventions.

[30] Philips Respironics (2004-2024). Actiware. Available from: https://www.usa.philips.com/healthcare/sites/actigraphy/solutions/actiware.

[31] Vandeleur M, Walter LM, Armstrong DS, Robinson P, Nixon GM, Horne RS (2017). How well do children with cystic fibrosis sleep? An Actigraphic and questionnaire-based study. J Pediatr..

[32] Tsai S-Y, Lee W-T, Jeng S-F, Lee C-C, Weng W-C (2019). Sleep and behavior problems in children with epilepsy. J Pediatr Health Care..

[33] Conley S, Knies A, Batten J, Ash G, Miner B, Hwang Y (2019). Agreement between actigraphic and polysomnographic measures of sleep in adults with and without chronic conditions: a systematic review and meta-analysis. Sleep Med Rev..

[34] Tétreault É, Bélanger M-È, Bernier A, Carrier J (2018). Actigraphy data in pediatric research: the role of sleep diaries. Sleep Med..

[35] Shrivastava D, Jung S, Saadat M, Sirohi R, Crewson K (2014). How to interpret the results of a sleep study. J Community Hosp Intern Med Perspect..

[36] Wilcox RR (1994). The percentage bend correlation coefficient. Psychometrika..

[37] Pike N (2011). Using false discovery rates for multiple comparisons in ecology and evolution. Methods Ecol Evol..

[38] R Core Team. A language and environment for statistical computing. 4.0.5 ed. Vienne, Austria2021.

[39] R Studio Team. RStudio: Integrated Development Environment for R. 1.4.1725 ed. Boston, MA2021.

[40] Akoglu H (2018). User's guide to correlation coefficients. Turk J Emerg Med..

[41] Knupp KG, Scarbro S, Wilkening G, Juarez-Colunga E, Kempe A, Dempsey A (2017). Parental perception of comorbidities in children with Dravet syndrome. Pediatr Neurol.

[42] Kaleyias J, Manley P, Kothare SV (2012). Sleep disorders in children with cancer. Semin Pediatr Neurol..

[43] Allen JM, Graef DM, Ehrentraut JH, Tynes BL, Crabtree VM (2016). Sleep and pain in pediatric illness: a conceptual review. CNS neurosci therapeutics..

[44] American Academy of Sleep Medicine. The International Classification of Sleep Disorders. 3rd ed. 2014. ISBN: 978-0-9915434-1-0; aasmnet.org.

[45] Tsai SY, Lee WT, Lee CC, Jeng SF, Weng WC (2018). Agreement between Actigraphy and diary-recorded measures of sleep in children with epilepsy. J nurs scholarship : an official public Sigma Theta Tau Int Honor Soc Nurs..

[46] Keilty K, Cohen E, Ho M, Spalding K, Stremler R (2015). Sleep disturbance in family caregivers of children who depend on medical technology: a systematic review. J Pediatr Rehabil Med..

[47] Lazzarin P, Schiavon B, Brugnaro L, Benini F (2018). Parents spend an average of nine hours a day providing palliative care for children at home and need to maintain an average of five life-saving devices. Acta Paediatr..

[48] Winger A, Kvarme LG, Løyland B, Kristiansen C, Helseth S, Ravn IH (2020). Family experiences with palliative care for children at home: a systematic literature review. BMC palliative care..

[49] Goldman SE, Bichell TJ, Surdyka K, Malow BA (2012). Sleep in children and adolescents with Angelman syndrome: association with parent sleep and stress. J Intellect Disabil Res..

[50] Werner H, Molinari L, Guyer C, Jenni OG (2008). Agreement rates between Actigraphy, diary, and questionnaire for Children's sleep patterns. Archiv pediat adoles med..

[51] Wayte S, McCaughey E, Holley S, Annaz D, Hill CM (2012). Sleep problems in children with cerebral palsy and their relationship with maternal sleep and depression. Acta paediatrica..

[52] Chen X, Gelaye B, Velez JC, Pepper M, Gorman S, Barbosa C (2014). Attitudes, beliefs, and perceptions of caregivers and rehabilitation providers about disabled children's sleep health: a qualitative study. BMC Pediatr..

[53] Galland BC, Short MA, Terrill P, Rigney G, Haszard JJ, Coussens S, et al. Establishing normal values for pediatric nighttime sleep measured by actigraphy: a systematic review and meta-analysis. Sleep. 2018;41(4) 10.1093/sleep/zsy017 29590464

[54] Surtees ADR, Richards C, Clarkson EL, Heald M, Trickett J, Denyer H (2019). Sleep problems in autism spectrum disorders: a comparison to sleep in typically developing children using actigraphy, diaries and questionnaires. Res Autism Spectr Disord..

[55] Dorris L, Scott N, Zuberi S, Gibson N, Espie C (2008). Sleep problems in children with neurological disorders. Develop neurorehab..

[56] Jan F (2004). Melatonin therapy for circadian rhythm sleep disorders in children with multiple disabilities: what have we learned in the last decade?. Dev Med Child Neurol..

[57] Merbler AM, Byiers BJ, Garcia JJ, Feyma TJ, Symons FJ (2018). The feasibility of using actigraphy to characterize sleep in Rett syndrome. J Neurodev Disord..

[58] Kirveskari E, Partinen M, Salmi T, Sainio K, Telakivi T, Hamalainen M (2000). Sleep alterations in juvenile neuronal ceroid-lipofuscinosis. Pediatr Neurol..

[59] Bautista M, Whittingham K, Edwards P, Boyd RN (2018). Psychometric properties of parent and child reported sleep assessment tools in children with cerebral palsy: a systematic review. Dev Med Child Neurol..

[60] Yavuz-Kodat E, Reynaud E, Geoffray M-M, Limousin N, Franco P, Bourgin P, et al. Validity of Actigraphy compared to polysomnography for sleep assessment in children with autism Spectrum disorder. Front Psychiatry. 2019:10. 10.3389/fpsyt.2019.00551 PMC6688709 31428003

[61] Xue B, Licis A, Boyd J, Hoyt CR, Ju Y-ES (2022). Validation of actigraphy for sleep measurement in children with cerebral palsy. Sleep Med..

