# Supplementary material for: Comparison of actigraphy with a sleep protocol maintained by professional caregivers and questionnaire-based parental judgment in children and adolescents with life-limiting conditions
Source: BMC Palliat Care. 2024 Feb 23;23:52. doi: 10.1186/s12904-024-01394-7 (PMC10885472; doi:10.1186/s12904-024-01394-7)
Supplement: Supplementary file 1 — Supplementary Material 1. [file 12904_2024_1394_MOESM1_ESM.pdf]

Supplementary file 1. Adapted version of the SCAC as used in the study (layout exclusive for this manuscript, does not correspond to the print version)

Sleep Screening for Children and Adolescents with Complex Chronic Conditions

# - SCAC -

DRAFT

Dear Parents,

Children and adolescents with serious illnesses frequently suffer from sleep problems.

Sleep problems can not only have a negative impact on a child's well-being: When a child sleeps poorly, it usually affects the whole family.

With your help, we can find out more about your child's sleep and possible sleep problems below.

There is no right or wrong way to answer the questions. Please choose the answer option that you think best applies to you or your child - you are the experts for your child!

Before you start

Please create your personal code for this questionnaire as follows:

- First digit: The **first** letter of <u>your child's</u> first name
- Second digit: The **first** letter of <u>your child's</u> last name
- Third and fourth digits: the **first two** digits of <u>your child's</u> birthday
- Fifth and sixth digits: The **first two** digits of the month of <u>your child's</u> birth.

Example*: John Doe, birthday on 02/01/2005;* ***code: JD0102***

Your personal code: __ __ __ __ __ __

Part A: Sleep (problems) and wakefulness

The following questions refer to your child's **sleep habits** and **daytime behavior**.

First, we would like to know more about your child's sleeping and waking times <u>during the last 3 days</u>.

1. At what time did you usually put your child to bed in the evening during the last 3 days?	
a) … from Monday – Friday?	____ : ____
b) … on weekends?	____ : ____
Attention: Please always enter the following time spans in hours and minutes.<br>For example, if your child was awake for 30 minutes during the night, enter "0 hour/s and 30 minutes".<br>For example, if your child was awake for 1.5 hours during the night, enter "1 hour/s and 30 minutes".	
2. How long has your child slept, on average, per night in total during the <u>last 3 days</u>?<br><br>*Note: If your child slept several times during the day and was awake in between, please add up the times your child actually slept.*<br>*Example: 5 hours awake - 1 hour sleep - 2 hours awake - 1.5 hours sleep = 2.5 hours sleep*	_____ hour/s and _____ minute/s
3. How long was your child awake, on average, per night in total during the <u>last 3 days</u>?<br><br>*Note: If your child has fallen asleep again in between, please add up the times your child was actually awake.*<br>*Example: 3 hours of sleep - 1 hour of wakefulness - 15 minutes of sleep - 2 hours of wakefulness = 3 hours of wakefulness.*	_____ hour/s and _____ minute/s
4. How often did your child wake up, on average, per night in total during the <u>last 3 days</u>?	____ times
5. At what time did your child usually wake up, or was woken up, in the morning during the <u>last 3 days</u>?	
c) … from Monday – Friday?	____ : ____
d) … on weekends?	____ : ____
6. How long has your child slept, on average, during the daytime in total during the last <u>3 days?</u>	_____ hour/s and _____ minute/s

DRAFT

The following questions refer to the situation in the **evening** and **at night.**

Please estimate on how many of the last 3 days a described situation occurred from your personal point of view.

Please always select only one answer option.

**1. On how many of the last 3 days...**	On...			
	no day	1 day	2 days	3 days
1a… did it take more than 20 minutes until your child fell asleep?				
1b. ... did your child fall asleep too early (more than 1 hour before the desired time)?				
1c. … did your child fall asleep too late (more than 1 hour after the desired time)?				
1d... did your child need support from you or another person to fall asleep (e.g., falling asleep together in one bed, rocking, holding hands, singing)?				
1e... did your child need support from anything else to fall asleep (e.g. nightlight, cuddly toy, radio)?				
1f... did your child wake up by itself (without any recognizable "disturbing influences") during the night?				

The following questions refer to the situation in the **morning** and **during the day**.

Please estimate on how many of the last 3 days a described situation occurred from your personal point of view.

Please always select only one answer option.

2. How long has your child slept, on average, during the daytime in total during the last 3 days?<br><br>*Note: If your child slept several times during the day and was awake in between, please add up the times your child actually slept.*<br>*Example: 5 hours awake - 1 hour sleep - 2 hours awake - 1.5 hours sleep = 2.5 hours sleep*	_____ hour/s and _____ minute/s

**3. On how many of the last 3 days…**	On...			
	no day	1 day	2 days	3 days
3a… did your child seem tired during the day?				
3b… did your child seem limp during the day?				
3c… did your child seem irritable during the day?				
3d… did your child seem hyper during the day?				
3e… did your child have a strong urge to sleep during the day that was hard to suppress?				
3f... did your child sleep unplanned during the day (e.g. suddenly falling asleep)?				

DRAFT

The following questions are generally about your child's sleep, whether during the night or during the day.

Please estimate on how many of the last 3 days a described situation occurred from your personal point of view.

Please always select only one answer option.

**4. On how many of the last 3 days did your child exhibit the following abnormalities of breathing during sleep?**	On…			
	no day	1 day	2 days	3 days
4a. Snored?				
4b. Slept with mouth open?				
4c. Strained breathing?				
4d. Shallow breathing?				
4e. Breathing stops?				
4f. Drops in oxygen saturation (if the child is monitored with a saturation monitor)?				
4g. Woke up with shortness of breath?				

**5. On how many of the last 3 days did your child exhibit the following behaviors during sleep?**	On…			
	no day	1 day	2 days	3 days
5a. Head was hyperextended backwards?				
5b. Appeared to be in a state between wakefulness and sleep?				
5c. Punched or kicked around?				

**6. On how many of the last 3 days did your child exhibit the following movements during sleep?**	On…			
	no day	1 day	2 days	3 days
6a. Movements that disturbed her/his sleep or falling asleep?				
6b. Repeated, rhythmic movements of the arms?				
6c. Repeated, rhythmic movements of the legs?				
6d. Repeated, rhythmic movements of other body parts (e.g. trunk, head)?				…
6e. Muscle twitching?				
6f. Any other noticeable movements (e.g. swinging of the head)?				

At the end of this first part, we would like to ask you a few general questions.

Please estimate on how many of the last 3 days a described situation occurred from your personal point of view.

Please always select only one answer option.

7. How would you describe your child's overall sleep quality?	Very good	Good	Satisfactory	Poor

8. Are you worried about your child's sleep?	Not at all	Somewhat	Quite	Very

DRAFT

Part B: Sleep circumstances

This second part of the questionnaire is about your child's sleep circumstances.

First, we would like to know more about the environment in which your child has slept during the last 3 days.

Please always select only one answer option.

**1. On how many of the last 3 days during the night was/were in your child's bedroom…**	On…			
	no day	1 day	2 days	3 days
1a… a light (e.g. ceiling light, night light) switched on?				
1b… environmental noises to be heard (e.g. street noise, monitor noise?				
1c… sounds being made by other persons to be heard (e.g. conversations)?				
1d… an electronic entertainment device switched on (e.g. Toni Box, TV, radio, tablet, computer)?				
1e… the door opened?				

The following questions are about your child's nighttime care during the last 3 days.

Please always select only one answer option.

**2. On how many of the last 3 days…**	On…			
	no day	1 day	2 days	3 days
2a… was your child repositioned at night?				
2b… was your child provided with assistive devices at night (e.g. orthoses)?				
2c… was your child ventilated at night?				
2d… was your child suctioned at night?				
2e… was your child nappy changed at night?				
2f… was your child given food or fluids at night?				
2g… was your child given medication/infusions at night?				

Lastly, we would like to ask you a few general questions about your child's symptoms at night and in the daytime during the last 3 days.

For each symptom, please indicate how often it occurred during the night and during the daytime.

Please always select only one answer option.

**3. On how many of the last 3 days during the night did your child experience...**	On…			
	no day	1 day	2 days	3 days
3a... pain?				
3b… irritability?				
3c… seizures?				
3d... severe spasticity?				
3e... nausea/vomiting?				
3f... constipation?				
3g... wounds/decubiti?				
3h... infections (e.g. flu-like infection)?				

DRAFT

	On...				
**4. On how many of the last 3 days during the daytime did your child experience...**	no day	1 day	2 days	3 days	
4a... pain?					
4b… irritability?					
4c… seizures?					
4d... severe spasticity?					
4e... nausea/vomiting?					
4f... constipation?					
4g... wounds/decubiti?					
4h... infections (e.g. flu-like infection)?					

Demographic part is not shown in this draft.

DRAFT
