# Supplementary material for: Comparison of actigraphy with a sleep protocol maintained by professional caregivers and questionnaire-based parental judgment in children and adolescents with life-limiting conditions
Source: BMC Palliat Care. 2024 Feb 23;23:52. doi: 10.1186/s12904-024-01394-7 (PMC10885472; doi:10.1186/s12904-024-01394-7)
Supplement: Supplementary file 2 — Supplementary Material 2. [file 12904_2024_1394_MOESM2_ESM.pdf]

Supplementary file 2. 24-hours sleep protocol

24-Stunden-Protokoll

Name:

Uhrzeit / Datum	6:00	7:00	8:00	9:00	10:00	11:00	12:00	13:00	14:00	15:00	16:00	17:00	18:00	19:00	20:00	21:00	22:00	23:00	0:00	1:00	2:00	3:00	4:00	5:00
Uhrzeit	6:00	7:00	8:00	9:00	10:00	11:00	12:00	13:00	14:00	15:00	16:00	17:00	18:00	19:00	20:00	21:00	22:00	23:00	0:00	1:00	2:00	3:00	4:00	5:00

erste Zeile: Schlafphasen (blau) | Wachphasen (grün) | Unruhe (rot) | Schreien (rot schraffiert) | Mahlzeiten (gelb)

zweite Zeile:

Ausmaß der Unruhe:
1 leicht 3 stark
2 mäßig 4 sehr stark

Weitere auftretende Symptome
S Schmerz Sp Spastik ⚡Krampfanfall
Dyspnoe Erbrechen/ Obstipation

Beispiele für mögliche Situationen
Mutter anwesend, Pflege durchgeführt, Musiktherapie, PEG,
Kanüle gewechselt, neues Gerät genutzt, neuer Rollstuhl, …
